# The Impact of Anti-Amyloid Immunotherapies on Stroke Care

**DOI:** 10.3390/jcm13051245

**Published:** 2024-02-22

**Authors:** Philippe A. Bilodeau, John R. Dickson, Mariel G. Kozberg

**Affiliations:** 1 Division of Neuroimmunology and Neuroinfectious Diseases, Massachusetts General Hospital, Harvard Medical School, Boston, MA 02114, USA; pbilodeau@mgh.harvard.edu; 2 MassGeneral Institute for Neurodegenerative Disease, Massachusetts General Hospital, Harvard Medical School, 114 16th Street, Charlestown, Boston, MA 02129, USA; john.dickson@mgh.harvard.edu; 3 J. Philip Kistler Stroke Research Center, Massachusetts General Hospital, Harvard Medical School, Boston, MA 02114, USA

**Keywords:** anti-amyloid immunotherapy, amyloid-related imaging abnormalities, ARIA-E, ARIA-H, cerebral amyloid angiopathy, ischemic stroke, antithrombotics, thrombolytics

## Abstract

Anti-amyloid immunotherapies have recently emerged as treatments for Alzheimer’s disease. While these therapies have demonstrated efficacy in clearing amyloid-β and slowing cognitive decline, they have also been associated with amyloid-related imaging abnormalities (ARIA) which include both edema (ARIA-E) and hemorrhage (ARIA-H). Given that ARIA have been associated with significant morbidity in cases of antithrombotic or thrombolytic therapy, an understanding of mechanisms of and risk factors for ARIA is of critical importance for stroke care. We discuss the latest data regarding mechanisms of ARIA, including the role of underlying cerebral amyloid angiopathy, and implications for ischemic stroke prevention and management.

## 1. Introduction

Anti-amyloid immunotherapies are the first disease-modifying treatments for Alzheimer’s disease (AD). Currently, there are two medications approved by the United States Food and Drug Administration (FDA) in this class: aducanumab and lecanemab. Additionally, donanemab recently demonstrated positive outcomes in a phase 3 clinical trial. The mechanism of action of these medications is antibody-mediated removal of amyloid-β (Aβ) from the brain. In large-scale clinical trials, patients receiving aducanumab, lecanemab, and donanemab had significant Aβ clearance and slower rates of cognitive decline [1,2,3,4,5]. Of note, other anti-amyloid immunotherapies discussed in this review have shown either a lack of Aβ clearance or lower than expected Aβ clearance, and the trials of these antibodies failed to show clinical benefit [6,7,8,9,10,11]. These outcomes suggest that clearance of fibrillar Aβ from the brain is a key aspect of the mechanism of action of anti-amyloid monoclonal antibodies since significant lowering of fibrillar Aβ as seen on amyloid positron emission tomography (PET) is seen only with those drugs that have achieved their primary endpoints in clinical trials. 

Anti-amyloid immunotherapies have been associated with amyloid-related imaging abnormalities (ARIA), which consist of two forms, ARIA-E (edema) and ARIA-H (hemorrhage), both of which are discussed in detail in this review. Given many patients with a diagnosis of AD have vascular risk factors and co-existent cerebrovascular disease, a critical question is whether using antithrombotics and thrombolytics is safe in patients receiving anti-amyloid immunotherapies. While the risks and benefits for each individual patient need to be carefully weighed, this review provides a comprehensive discussion of the known mechanisms of ARIA as well as the currently available data regarding the safety of these agents.

Approximately 50% of patients with AD have co-existent moderate-to-severe cerebral amyloid angiopathy (CAA) [12], an important risk factor for ARIA. CAA, a leading cause of lobar intracerebral hemorrhage in the elderly, is a cerebral small vessel disease in which Aβ is deposited within the walls of leptomeningeal and cortical blood vessels. It has been proposed that there is a significant mechanistic overlap between ARIA and CAA-related inflammation (CAA-ri), a condition in which patients develop auto-antibodies to Aβ resulting in clinically significant brain inflammation and hemorrhagic events [13]. Additionally, anti-amyloid immunotherapies may exacerbate vascular Aβ deposition [14,15,16]. Concerns about safety and efficacy in patients with a clinical diagnosis of CAA prompted a statement from experts in the field recommending against off-label use of aducanumab (at the time the only FDA approved anti-amyloid immunotherapy) for the treatment of CAA [17]. This review discusses the overlap between ARIA and CAA, including implications for secondary ischemic stroke prevention in patients at risk for ARIA.

## 2. Defining Amyloid-Related Imaging Abnormalities: Insights from Clinical Trials

Imaging abnormalities have been consistently recognized as a potential adverse effect of anti-amyloid immunotherapy. The first recognition of these adverse events was during a phase 1 trial of active immunization with pre-aggregated Aβ42 plus adjuvant (AN1792) which resulted in 18 cases of meningoencephalitis. These cases had a variety of magnetic resonance imaging (MRI) abnormalities, including fluid-attenuated inversion recovery (FLAIR) hyperintensities and leptomeningeal enhancement [18]. The MRI techniques used in this study may not have been sufficiently sensitive to detect microhemorrhages, but three participants experienced cerebral hemorrhages during the study [18]. Passive immunization strategies for anti-amyloid immunotherapy have also been associated with imaging abnormalities. In the phase 1 study of bapineuzumab, three participants receiving the highest dose developed evidence of vasogenic edema on the FLAIR sequence [19]. One of these participants also developed a new punctate magnetic susceptibility lesion on the gradient echo (GRE) sequence that was suspected to represent a microhemorrhage [19]. The development of vasogenic edema on MRI was also associated with bapineuzumab treatment in a phase 2 trial [20].

In response to these adverse events, a nomenclature was developed to describe them. These MRI changes associated with anti-amyloid immunotherapy have been termed amyloid-related imaging abnormalities (ARIA), and they are subdivided into two distinct phenomena: ARIA-E (edema), in which FLAIR hyperintensities are seen in the brain parenchyma or leptomeninges, and ARIA-H (hemorrhage), in which hemosiderin deposits in the form of microhemorrhages or cortical superficial siderosis (cSS) are seen on GRE or susceptibility weighted imaging (SWI) [21]. In subsequent clinical trials, including phase 3 trials, ARIA has been observed consistently as an adverse effect of different anti-amyloid immunotherapies. While frequently asymptomatic, clinical symptoms of ARIA-E can include headaches, confusion, vision changes, gait disturbances, focal neurological deficits, and even death [3,4,5,6,22,23]. ARIA-H is typically asymptomatic but occasionally is associated with symptomatic intracerebral hemorrhage; specific examples of these events in recent trials are discussed further below. A framework for grading the radiologic severity of ARIA is presented in Table 1 [3,21]. Ultimately, ARIA has emerged as an important consideration for both clinical trial design and clinical care with anti-amyloid immunotherapy.

The working group who defined ARIA also proposed recommendations for clinical trials, including MRI protocol minimum standards, MRI monitoring for the development of ARIA, MRI reading and reporting, and exclusionary findings [21]. For enrolling participants in an anti-amyloid immunotherapy trial, a cut-off of four microhemorrhages on baseline MRI was recommended for exclusion to reduce the risk for ARIA-H. Monitoring for ARIA with regular MRIs including T2-FLAIR and hemosiderin-sensitive sequences was recommended, and discontinuation of therapy with incident ARIA-H was recommended for participants with significant clinical symptoms or evidence of a precipitous clinical decline [21]. In addition to these surveillance scans, MR imaging is recommended for patients with clinical symptoms suggestive of ARIA-E. For the two anti-amyloid antibodies to receive accelerated or full approval by the United States Food and Drug Administration (FDA), aducanumab [24] and lecanemab [25], the phase 3 clinical trials excluded patients with more than four microhemorrhages and/or a finding of cSS on MRI [3].

While the accumulated evidence from clinical trials of anti-amyloid immunotherapy points to a causal relationship between these therapies and ARIA, trials performed in larger cohorts (especially phase 3 trials) have shown evidence of both ARIA-E and ARIA-H in the placebo group, but generally at a lower rate than in the treatment group [3,4,5,6,7,26,27] (Table 2). In this context, it is important to recognize that ARIA is a radiographically defined entity [21] and does not necessarily describe specific pathological entities or pathophysiologic processes. The development of “spontaneous ARIA”, also termed cerebral amyloid angiopathy-related inflammation (CAA-ri), has also been reported outside of the context of anti-amyloid immunotherapy trials [28,29], and is discussed further below. Taken together, these data suggest that the imaging findings in ARIA may represent a spectrum of disease encompassing spontaneously occurring CAA/CAA-ri and anti-amyloid immunotherapy-induced edema or hemorrhage.

## 3. Risk Factors for ARIA

Clinical trial data have revealed risk factors for ARIA (summarized in Table 3), providing some insights into potential underlying mechanisms. Given the occurrence of ARIA as adverse events associated with several different anti-amyloid antibodies [3,4,5,6,22], the risk of ARIA seems to be related to this general class of drugs. The notable exceptions to this are solanezumab and crenezumab, which have consistently shown no increased risk of ARIA compared to placebo. In two phase 3 trials of solanezumab in mild-to-moderate Alzheimer’s disease, there was no significant difference in ARIA-E or ARIA-H rates between solanezumab- and placebo-treated participants [7]. In a placebo-controlled trial of solanezumab or gantenerumab in participants with dominantly inherited Alzheimer’s disease, solanezumab was not associated with an increased risk of ARIA but gantenerumab was [22]. In a phase 3 trial of solanezumab in preclinical Alzheimer’s disease, there was no difference in ARIA rates between solanezumab and placebo [30]. Two phase 3 clinical trials of crenezumab in prodromal to mild Alzheimer’s disease showed no difference in ARIA rates between crenezumab and placebo [10]. However, notably neither of these antibodies demonstrated significant clinical benefit in these trials [7,8,9,10]. Additionally, neither solanezumab nor crenezumab were found to significantly lower fibrillar Aβ as seen on amyloid positron emission PET scans conducted in biomarker substudies within these trials [7,8,9,10]. The lack of amyloid clearance resulting from treatment with solanezumab and crenezumab is a possible explanation for why these antibodies have neither clinical effectiveness in treating AD nor increased risk of developing ARIA. However, the mechanism for this is not fully elucidated. It is notable that both solanezumab and crenezumab share a similar epitope in the mid-region of Aβ [27], meaning the antibodies bind to a similar region of Aβ. This may contribute to the fact that both antibodies bind monomeric Aβ, but small differences in the epitopes may explain why solanezumab binds preferentially to Aβ monomers and crenezumab also binds Aβ oligomers [31]. While the similar binding region and affinity for Aβ monomers are a possible explanation for the lack of amyloid clearance, clinical effectiveness, and ARIA risk with these drugs, further research is needed to fully elucidate the mechanisms underlying these findings. Additionally, crenezumab was developed to minimize FcγR activation [31], and the lack of ARIA associated with this antibody suggests that complement activation may be part of the immune response leading to ARIA.

Another risk factor for the development of ARIA is a higher dose of anti-amyloid antibody. This relationship was suggested in the phase 1 trial of bapineuzumab in which the imaging changes now called ARIA were only seen in the highest dose of this dose-escalation study [19]. This dose-dependence was also seen in the phase 2 trial [20]. A dose-dependent increase in ARIA rates was also observed in trials of gantenerumab [26,32], aducanumab [2,3], lecanemab [1], and to some degree donanemab [33]. 

The time course of treatment also seems to be associated with ARIA risk, with multiple studies showing higher ARIA risk earlier in the treatment course and lower risk later in the treatment course [26,34,35,36,37]. The mechanism underlying this temporal association with ARIA risk is unclear. One proposed hypothesis is that initial treatment leads to a loss of blood vessel integrity, which increases the risk for ARIA, but subsequent vascular remodeling over time decreases the risk for ARIA [38].

The APOE ε4 allele has consistently been associated with increased ARIA risk [3,4,5,6,26]. This association has implications for clinical care, with appropriate use recommendations for aducanumab indicating APOE genotyping should be considered when it could influence patients’ decisions regarding therapy [39]. The appropriate use recommendations for lecanemab recommend APOE genotyping for all potential treatment candidates [40]. The FDA label for lecanemab includes a boxed warning regarding the risk for ARIA and indicates that APOE genotyping should be performed prior to initiation of treatment [41].

In addition to being a risk factor for ARIA, the APOE ε4 allele is also a risk factor for Alzheimer’s disease [42], CAA [43], and CAA-ri [44]. The shared association between the APOE ε4 allele and ARIA or CAA risk is particularly notable given the similar imaging characteristics of these two entities. Both ARIA-H and CAA exhibit microhemorrhages and/or cSS on imaging [21,45]. The FLAIR hyperintensities seen in ARIA-E resemble the MRI changes seen with CAA-ri [21,46]. Additionally, imaging markers consistent with CAA including microhemorrhages and cortical superficial siderosis have been demonstrated to be independently associated with higher ARIA-E risk in donanemab trials [47]. Together, these similar genetic risks and imaging characteristics hint at possible shared underlying pathophysiologic mechanisms in ARIA and CAA, discussed further below.

## 4. Cerebral Amyloid Angiopathy-Related Inflammation: Spontaneous ARIA?

**Figure 1:**
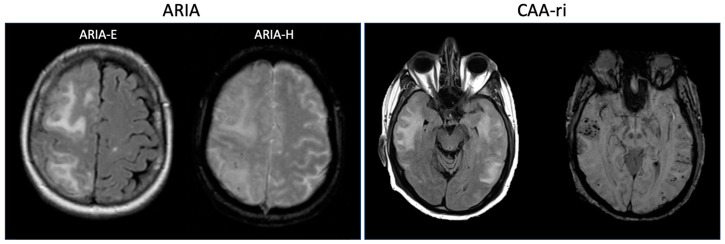
(**Left**): Representative images from a patient with concomitant ARIA-E and ARIA-H (reproduced with permission from Sperling et al., 2011 [21]). The T2-FLAIR image on the left demonstrates regions of edema (ARIA-E) in the right frontal and parietal lobes, and the GRE image on the right demonstrates microhemorrhages (ARIA-H) in the right parietal lobe. (**Right**): Representative images from a patient with CAA-ri. The T2-FLAIR image on the left demonstrates edema in bilateral temporal lobes, and the SWI image on the right demonstrates multiple microhemorrhages within these regions.

CAA-ri is a syndrome characterized clinically by acute or subacute encephalopathy, headaches, seizures and focal neurologic deficits and radiographically by evidence of asymmetric FLAIR hyperintensities and associated microhemorrhages on GRE or SWI sequences [48], similar in appearance to ARIA-E and ARIA-H, respectively (Figure 1). It was first described in 2004 by Eng and colleagues, who found that 7/42 patients with pathologically diagnosed CAA had evidence of perivascular inflammation [49]. These 7 patients all had encephalopathy and 71% had APOE ε4/ε4 alleles. Further studies have confirmed an association with the APOE ε4 allele [44]. Chung and colleagues proposed diagnostic criteria for CAA-ri [46] which were further refined by Auriel and colleagues [50]. Probable CAA-ri requires age ≥ 40 years old, presence of ≥1 of the following clinical features: headache, decrease in consciousness, behavioral change, or focal neurological signs and seizures, MRI with unifocal or multifocal WMH lesions (corticosubcortical or deep) that are asymmetric and extend to the immediately subcortical white matter and presence of ≥1 of the following corticosubcortical hemorrhagic lesions: cerebral microhemorrhage or cortical superficial siderosis. These updated criteria were found to have a sensitivity and specificity of 82% and 97%, respectively, when compared to pathologically confirmed CAA-ri [50]. Cerebrospinal fluid analysis in CAA-ri generally shows mild pleocytosis and elevated protein [51].

**Figure 2:**
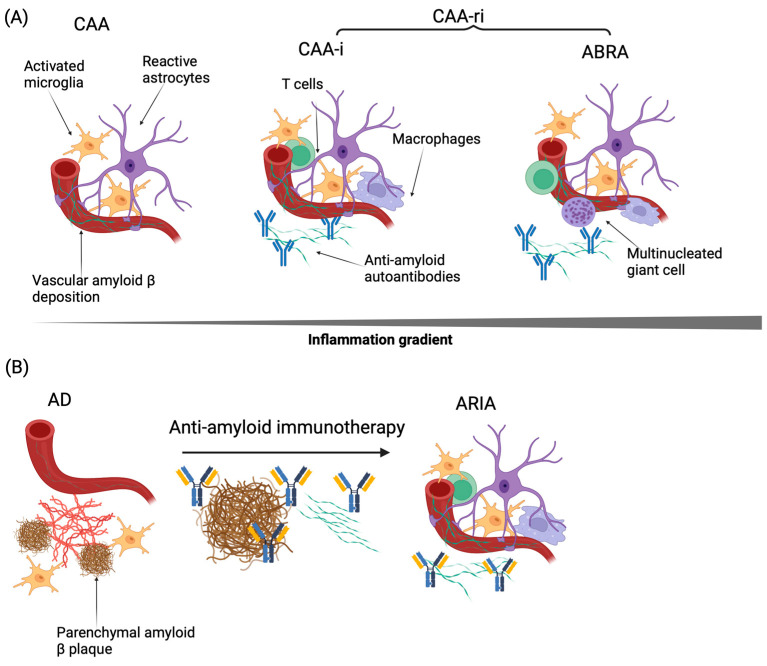
Inflammatory profiles associated with CAA, CAA-ri, AD, and ARIA. (**A**) CAA, CAA-i, and ABRA represent a spectrum of perivascular and transmural inflammation. CAA-ri (including CAA-i and ABRA) has been associated with anti-amyloid autoantibodies. (**B**) AD has been associated with subclinical inflammation, and ARIA, observed either spontaneously or in the context of anti-amyloid immunotherapy, involves more substantial perivascular inflammation. These anti-amyloid antibodies may both mobilize Aβ to the vasculature (exacerbating CAA burden) and additionally remove Aβ from the vasculature through inflammatory mechanisms, damaging the vasculature.

Pathologically, CAA-ri is associated with leptomeningeal and cortical monocytic infiltrates, and additionally shows memory T cell and CD8+-predominant T cells infiltrates [52]. There is also evidence of microglial activation by PET [53]. In some severe cases, pathology shows a granulomatous angiitis characterized by angiodestructive inflammation within the vessel wall, with lymphocytic infiltration and fibrinoid necrosis [54,55]. This pathological phenotype has been termed Aβ-related angiitis (ABRA). Clinically, patients with ABRA are more likely to have infarcts on brain MRI and have presentations more closely resembling primary angiitis of the central nervous system (PACNS) [55,56]. Consequently, ABRA is thought to represent one end of the CAA-ri spectrum, with inflammatory CAA (CAA-i) representing the milder end. Interestingly, sporadic “non-inflammatory” CAA cases also show evidence of blood brain barrier breakdown and perivascular inflammation; suggesting there may be a spectrum of inflammation in CAA, CAA-i, and ABRA [57] (Figure 2).

Immunologically, patients with CAA-ri have anti-Aβ40 and Aβ42 autoantibodies in the acute phase, and antibody levels are correlated with levels of Aβ40 and Aβ42 [13,58]. Studies using Florbetapir-PET, which uses a radionuclide that binds to Aβ, have demonstrated lower cortical tracer uptake in areas of prior inflammation, suggesting that Aβ removal from the brain may be involved in the disease [59]. The presence of autoantibodies to Aβ and the suggestion of Aβ removal in CAA-ri both notably parallel the mechanism of action of anti-amyloid immunotherapies.

Additionally, the vascular inflammation associated with anti-amyloid immunotherapy-related ARIA is similar to CAA-ri (Figure 2). Some patients in the early amyloid active immunization trials had a lymphocytic meningoencephalitis with macrophage and microglial infiltration [60]. While the newer anti-amyloid immunotherapies do not typically cause meningoencephalitis, they have been associated with vascular inflammation, including peripheral immune cell infiltration. Indeed, the autopsy of a patient who received lecanemab and had multiple tissue plasminogen activator (tPA)-associated intraparenchymal hemorrhages (discussed further below), revealed a histiocytic vasculitis with fragmentation and phagocytosis of vascular Aβ [23,61]. Additionally, a recent autopsy of a patient with severe lecanemab-induced symptomatic ARIA demonstrated lymphocytic infiltrates, macrophages, multinucleated giant cells, and meningeal vessels and penetrating arterioles with Aβ deposition and evidence of fibrinoid necrosis [62]. Notably, both cases had evidence of severe inflammation within Aβ-laden vessel walls, resembling ABRA as discussed above.

The pathophysiology of ARIA remains unclear but given radiographic and pathologic overlap with CAA-ri and shared risk factors, many hypothesize that vascular Aβ is a major contributor to ARIA [15,16]. One potential mechanism is that in the process of clearance of parenchymal Aβ by anti-amyloid immunotherapies, vascular Aβ accumulates (Figure 2). This is supported by a neuropathological study which demonstrated increased Aβ42, the peptide typically associated with parenchymal Aβ plaques in AD, within the walls of cerebral blood vessels and leptomeninges in patients who had received active immunization against Aβ42 with AN1792 [14]. This finding is particularly significant as Aβ40 is the peptide more commonly observed in vascular amyloid deposits. Additionally, in two patients whose brains were examined histopathologically 4–5 years after immunotherapy with AN1792, an absence of both parenchymal and vascular Aβ was observed. Together, these findings suggest that the initial removal of parenchymal Aβ42 may paradoxically exacerbate CAA, and that subsequent vascular amyloid clearance occurs. Further suggesting that CAA may underlie ARIA mechanisms, a separate neuropathological study of patients who received AN1792 examined regions with cSS, a marker of ARIA-H, observing a strong relationship between cSS and leptomeningeal CAA with concentric vessel wall splitting [63]. A likely further contributing mechanism to ARIA is that the removal of vascular Aβ by anti-amyloid immunotherapies leads to vascular damage, in turn leading to blood-brain barrier leakage (and edema, ARIA-E) as well as blood vessel rupture (and hemorrhage, ARIA-H), directly echoing the proposed mechanisms of CAA-ri [16]. Supporting this hypothesis, a recent study investigating ARIA in a mouse model of AD using the murine equivalent of the anti-amyloid antibody bapineuzumab, 3D6, demonstrated that administering anti-amyloid antibodies triggered the formation of antibody immune complexes with vascular Aβ, in turn activating perivascular macrophages and upregulating genes associated with vascular permeability [64]. Antibody-mediated complement activation may be an important step in immune cell activation leading to vascular damage and ARIA [65,66]. Modifying these antibodies to minimize complement activation is an area of active investigation.

There is limited data or guidance regarding the treatment of ARIA, and additionally no randomized data regarding the treatment of CAA-ri. For asymptomatic cases of ARIA, holding, decreasing the dose, or discontinuing the drug may be sufficient. For symptomatic cases of ARIA-E, treatment with steroids is generally advised. Similarly, the mainstay of treatment for CAA-ri is high-dose corticosteroids. In select cases of CAA-ri, additional steroid-sparing immunotherapy such as mycophenolate, methotrexate, cyclophosphamide, and intravenous immunoglobulins (IVIG) are considered [67]. Specifically, in the case of ABRA, cyclophosphamide is often used given the severity of the disease [55,56]. Treatment of CAA-ri is associated with clinical and radiological improvement and reduces the likelihood of recurrence [28,68]. Over 70% of CAA-ri patients recover clinically within 3 months of their first presentation of CAA-ri, while radiological recovery can take up to 12 months [28]. Of note, while the edema in CAA-ri and ARIA-E may improve or resolve with treatment, the hemorrhagic markers associated with CAA-ri and ARIA-H (microhemorrhages and cSS) will remain on repeat scans.

## 5. Secondary Ischemic Stroke Prevention in Patients Receiving Anti-Amyloid Immunotherapy

Given the potential risks of exacerbating or triggering ARIA-H, the safety of using long-term antithrombotics in patients who are receiving anti-amyloid immunotherapy is a concern. There is minimal data available thus far, and therefore individual risk/benefit assessments and shared decision-making is essential. Given the significant clinical overlap between ARIA-H and CAA, data regarding ICH risk reduction in patients with CAA may be relevant for patients receiving anti-amyloid immunotherapies and will also be discussed below. Of note, patients with a history of recent stroke or TIA (within 12 months of screening) and/or evidence of chronic cortical or lacunar infarcts were excluded from the recent phase 3 studies of aducanumab and lecanemab (but not excluded from the phase 3 donanemab trial) [3,4,5], somewhat limiting the applicability of their data to a high ischemic stroke-risk population. Additionally, many phase 3 trials of anti-amyloid immunotherapies excluded patients with evidence of severe small vessel disease. Table 4 details cerebrovascular-related exclusion criteria for each phase 3 trial of passive anti-amyloid immunotherapies, including trials of bapineuzumab, solanezumab, gantenerumab, crenezumab, aducanumab, lecanemab, and donanemab.

Key features to consider when assessing ARIA-H risk in an individual patient (and deciding on subsequent antithrombotic therapy) include the following: (1) the timeline of initiation of anti-amyloid immunotherapy, (2) presence of APOE ε4 alleles, and (3) pre-existing cerebral microhemorrhages and/or cSS. Data from the recent phase 3 study of lecanemab suggest that ARIA-H frequently occurs with ARIA-E, a finding observed in phase 3 studies of aducanumab and donanemab as well [3,4]. As discussed above, the risk of ARIA-E is highest immediately after treatment initiation, and likewise instances of co-occurring ARIA-E and ARIA-H are typically observed within the first 6 months of therapy initiation. Therefore, ideally one would avoid antithrombotics throughout this higher ARIA risk period. Of note, in the lecanemab trial, isolated ARIA-H did occur throughout the study period, both in patients receiving lecanemab (8.9%) and placebo (7.8%) [5]. Similarly, in the donanemab trial, the incidence of isolated ARIA-H was comparable across the donanemab (12.7%) and placebo (12.4%) groups [4]. These isolated ARIA-H events may reflect, in part, underlying CAA disease progression, unrelated to drug administration.

When considering an individual patient’s ARIA-H/ICH risk related to underlying CAA, multiple clinical and radiographic factors are relevant. cSS has recently emerged as one of the most important predictors of subsequent ICH in patients with CAA [69,70,71,72], and patients with a history of ICH and disseminated cSS are at highest risk for subsequent intracerebral hemorrhage with one study reporting annual hemorrhage rates up to 27% [72]. Other risk factors for subsequent ICH include age, microhemorrhage number, and APOE allele status (with both APOE ε2 and APOE ε4 being risk factors for hemorrhage in CAA, differing from ARIA in which only APOE ε4 is a risk factor) [73]. 

### 5.1. Antiplatelets

Aspirin use was allowed in all phase 3 trials of passive anti-amyloid immunotherapies; however, the use of other antiplatelet agents was restricted in the phase 3 trial of aducanumab (Table 4). Data from the phase 3 trial of lecanemab demonstrated lower rates of ARIA-E, microhemorrhages, and cSS in patients receiving antiplatelet therapy and lecanemab rather than lecanemab alone [5] suggesting relative safety of these agents.

Antiplatelet use is typically limited in patients with CAA to those who have established indications. The Restart or STop Antithrombotics Randomised Trial (RESTART) randomized patients with a recent ICH (either lobar or deep) who were on antiplatelet therapy for secondary prevention to either restart or discontinue aspirin and found no difference in ICH recurrence between the two groups [74]. Based partly on this reassuring data, the 2022 American Heart Association guidelines state that it is reasonable to resume antiplatelet therapy after ICH in patients with an established indication (Class 2b) [75].

Given this data from patients with a history of ICH and reassuring data from the lecanemab phase 3 trial mentioned above, it is reasonable to consider using antiplatelet medications in patients at risk for ARIA when there is a clear clinical indication. However, the risks/benefits of antiplatelet agents, including dual antiplatelet therapy which was used only in a minority of patients in these trials, needs to be weighed on an individualized basis.

### 5.2. Anticoagulation

Concomitant treatment with anticoagulants was exclusionary in aducanumab [3] but not lecanemab or donanemab trials [4,5] (Table 4). Recently, details on adverse events in the phase 3 trial of lecanemab and open-label extension were published, stratified by patients who received therapeutic anticoagulation or tPA [76]. While the numbers are limited, patients receiving lecanemab and anticoagulation or tPA (140 patients in total) had higher rates of macrohemorrhage than those receiving lecanemab and not receiving anticoagulation/tPA (3.6% vs. 0.3%). These numbers include two patient deaths in the open-label extension which are discussed further below. Of note, there were 3 ARIA-related deaths reported in the donanemab trial, and all 3 of these occurred in patients who were not on antithrombotics and had not received thrombolytics [4]. Given the currently available data, current published appropriate use recommendations for both aducanumab and lecanemab recommend against initiating anti-amyloid immunotherapy in patients receiving anticoagulation until more data are available [39,40].

Because of the elevated risk of CAA-related ICH with long-term anticoagulation, many consider alternatives to anticoagulation in patients with CAA, particularly in patients with high-hemorrhage risk features [73]. One of the most common indications for long-term anticoagulation is non-valvular atrial fibrillation, and the risks and benefits of anticoagulation for atrial fibrillation in patients with a history of ICH is an area of active study. Three recently published randomized clinical trials addressing this question, NASPAF-ICH, SoSTART, and APACHE-AF, as well as a recent meta-analysis, were inconclusive [74,77,78,79]. There are several ongoing studies aiming to answer this question including ASPIRE (NCT03907046), ENRICH-AF (NCT03950076), A3ICH (NCT03243175), and PRESTIGE-AF (NCT03996772). Note, the ENRICH-AF data safety monitoring board (DSMB) recently performed a safety review of the first 699 enrollments and recommended that patients with lobar ICH or convexity subarachnoid hemorrhage stop receiving the study drug and that no further patients should be enrolled with these types of hemorrhage [80]. This patient population is thought to primarily represent patients with CAA, further suggesting caution with the use of anticoagulation in a patient population with CAA.

Left atrial appendage closure (LAAC) has been shown in recent trials to be non-inferior to anticoagulation for ischemic stroke prevention in the setting of non-valvular atrial fibrillation [81,82,83], and is a possible alternative to long-term anticoagulation in patients with CAA. A recent observational cohort study also suggested safety of this procedure in a patient population with CAA [84]. However, it is important to note that LAAC has not yet been tested in a randomized clinical trial in a CAA/ICH patient population. As LAAC currently requires higher doses of antithrombotics peri- and post-procedurally, one might consider pursuing LAAC and completing the post-procedural higher dose antithrombotic period prior to initiation of anti-amyloid immunotherapy in patients with non-valvular atrial fibrillation.

Of note, depending on the indication, the benefits of short-term anticoagulation may exceed the risks in patients with CAA, even in particularly high-hemorrhage risk patients. This may also be a consideration for patients receiving anti-amyloid immunotherapies, particularly those patients who are past the initial high ARIA-risk period, based on the clinical scenario.

### 5.3. Hypertension and Hyperlipidemia Management

While limited data exists regarding blood pressure management in the setting of anti-amyloid immunotherapies, hypertension management is one of the most important modifiable risk factors in reducing the risk of CAA-related ICH [73], and is also a mainstay of ischemic stroke prevention. Recently presented work also suggested that elevated mean arterial pressure was independently associated with a higher risk of ARIA-E with donanemab [47]. Current guideline recommendations are to maintain blood pressures less than 130/80 long-term in patients at risk for ICH [75], and some providers target a blood pressure goal of less than 120/80 in patients with a diagnosis of CAA.

Hyperlipidemia management is also an essential component of secondary ischemic stroke prevention. However, a post-hoc analysis from the Stroke Prevention by Aggressive Reduction in Cholesterol Levels (SPARCL) trial demonstrated a higher risk of recurrent ICH in patients with a history of ICH who received atorvastatin [85]. Other observational and Mendelian randomization studies have also suggested an inverse association between LDL levels and ICH risk [86,87]. However, some observational studies including a recent population-based, propensity score-matched cohort study from Denmark, have not demonstrated an increased risk of ICH in people receiving statins [88]. The ongoing Statins in Intracerebral Hemorrhage (SATURN) trial (NCT03936361) which is enrolling patients with a history of lobar ICH while on a statin is expected to provide further information regarding the effects of continuing vs. discontinuing statins in patients with CAA. For now, given the established cardiovascular benefits of statins, it is reasonable to consider continuing statins in patients with CAA or receiving anti-amyloid immunotherapies who have an established indication per ACC/AHA guidelines. In these cases, each patient’s individual risk of ischemic cardiovascular and cerebrovascular events should be considered versus ICH risk [75].

## 6. Acute Stroke Therapy and Emergent Anticoagulation in Patients Receiving Anti-Amyloid Immunotherapy

Whether thrombolytics and/or acute anticoagulation exacerbate ARIA-H risk has not been explicitly studied in the recent phase 3 trials of anti-amyloid immunotherapies. In emergent situations such as acute stroke or myocardial infarction in a patient already on anti-amyloid immunotherapy, case-by-case multidisciplinary discussions of risks and benefits of thrombolytics or anticoagulation will be needed. As mentioned above, two patient deaths secondary to ICH occurred in the open label extension of the phase 3 trial of lecanemab. One of these patients had received tPA [23] for suspected ischemic stroke [23] and the other had received heparin for a myocardial infarction.

Considering acute anticoagulation, based on data suggesting increased ARIA-H risk associated with anticoagulation in patients receiving anti-amyloid immunotherapy, it may be reasonable to perform urgent advanced imaging (e.g., MRI) in an acute setting when considering the risks and benefits of emergent anticoagulation to ensure there is no ongoing ARIA (e.g., edema suggesting ongoing ARIA-E and/or new microhemorrhages/cSS suggesting recent/ongoing ARIA-H). However, more data are needed to determine if this is necessary and/or sufficient.

Regarding thrombolysis, whether the use of anti-amyloid immunotherapy should be considered a relative or absolute contraindication is an area of active debate. Autopsy findings from the patient who received tPA while on lecanemab demonstrated significant vascular inflammation [23], suggesting ARIA-E was likely present at the patient’s initial presentation. Given the overlap between ARIA-E and ARIA-H, one can consider this as a contributing factor to the patient’s subsequent ICHs which were more severe and multifocal than is typically observed with post-tPA hemorrhagic transformation. Based in part on this case, current appropriate use recommendations for lecanemab recommend against the use of thrombolytics until additional safety evidence is available [40].

Note, limited data is available regarding whether the benefits of thrombolysis outweigh the risks in patients with CAA. While retrospective studies have demonstrated that patients with microbleeds (particularly > 10 microbleeds) are at higher risk of symptomatic ICH post-tPA [89,90], it remains unclear whether the potential benefits of thrombolysis outweigh these risks. Current guidelines from the American Heart Association acknowledge the increased risk of symptomatic ICH post-thrombolysis in a patient population with a high burden of microbleeds and state that if a patient has > 10 microbleeds on MRI “treatment may be reasonable if there is the potential for substantial benefit” (IIb) [91].

Endovascular thrombectomy may be a reasonable acute treatment option for patients on anti-amyloid immunotherapy with an accessible lesion. However, if there is clinical uncertainty about the presence of ongoing ARIA-E (e.g., edema on initial imaging, known APOE ε4 genotype, recent initiation of anti-amyloid immunotherapy), one may also consider advanced imaging (e.g., MRI) prior to the procedure. While there is currently no available data in support of this, patients with ongoing ARIA could be at higher risk for reperfusion injury.

## 7. Conclusions

The anti-amyloid immunotherapies are newly FDA-approved, disease-modifying therapies for AD. This class of medications has been associated with ARIA-E and ARIA-H, side effects which have been mechanistically linked to the cerebral vasculature, and specifically to CAA. As anti-amyloid immunotherapies begin to be prescribed more broadly, an understanding of the risk factors for, mechanisms of, and monitoring strategies for ARIA will be critical for both stroke prevention and management in patients receiving anti-amyloid immunotherapies. Ongoing studies focused on understanding the pathophysiological mechanisms underlying ARIA may aid in further defining ARIA risk factors and prevention strategies.

## Figures and Tables

**Table 1:** Radiographic Classification of ARIA Severity.

	Mild	Moderate	Severe	Location of Increased Vascular Permeability	
				Parenchyma	Leptomeninges
ARIA-E	FLAIR hyperintensity confined to sulcus and or cortex/subcortical white matter in one location < 5 cm	FLAIR hyperintensity 5 to 10 cm, or more than 1 site of involvement, each measuring <10 cm	FLAIR hyperintensity measuring >10 cm, often with significant subcortical white matter/sulcal involvement. May involve one or more separate sites	“Vasogenic edema”	Sulcal effusion/exudate
ARIA-H	≤4 new microhemorrhages on T2*-GRE OR 1 focal area of superficial siderosis on T2*-GRE	5 to 9 new microhemorrhages OR 2 focal areas of superficial siderosis	10 or more new microhemorrhages OR >2 focal areas of superficial siderosis	Microhemorrhages	Superficial hemosiderosis

**Table 2:** Summary of ARIA in Phase 3 Clinical Trials of Passive Anti-Amyloid Immunotherapy in Symptomatic Alzheimer’s Disease.

	Bapineuzumab [6]	Solanezumab [7,8]	Gantenerumab [11,26]	Crenezumab [10]	Aducanumab [3]	Lecanemab [5]	Donanemab [4]
ARIA-E rate							
Treatment arm	15.3% (APOE ε4 carrier study); 4.2–14.2% (APOE ε4 noncarrier study)	0.9% [7]; 1% [8]	6.6% (105 mg dose) [26]; 13.5% (225 mg dose) [26]; 24.9% (pooled) [11]	0.3% (CREAD and CREAD2)	26–35% (EMERGE); 26–36% (ENGAGE)	12.6%	24.0%
Placebo arm	0.2% (APOE ε4 carrier study); 0.2% (APOE ε4 noncarrier study)	0.4% [7]; 2% [8]	0.8% [26]; 2.7% (pooled) [11]	0.3% (CREAD); 0% (CREAD2)	2% (EMERGE); 3% (ENGAGE)	1.7%	1.9%
ARIA-H rate							
Treatment arm	Not reported	4.9% [7]; 3.5% [8]	22.9% (105 mg dose) [26]; 16.2% (225 mg dose) [26]; 22.9% (pooled) [11]	9.8% (CREAD); 5.0% (CREAD2)	10–20% (EMERGE); 9–19% (ENGAGE)	17.3%	19.7%
Placebo arm	Not reported	5.6% [7]; 2.8% [8]	13.2% [26]; 12.3% (pooled) [11]	7.8% (CREAD); 5.9% (CREAD2)	7% (EMERGE); 6% (ENGAGE)	9.0%	7.4%

**Table 3:** ARIA Risk Factors.

Anti-amyloid antibody treatment (except solanezumab and crenezumab)
Higher anti-amyloid antibody dose
Early timepoint in treatment course (especially first 6 months)
Presence of APOE ε4 allele
Underlying cerebral amyloid angiopathy

**Table 4:** Summary of Cerebrovascular-Related Exclusions of Phase 3 Clinical Trials of Passive Anti-Amyloid Immunotherapy in Symptomatic Alzheimer’s Disease.

	Bapineuzumab [6]	Solanezumab [7,8]	Gantenerumab [11,26]	Crenezumab [10]	Aducanumab [3]	Lecanemab [5]	Donanemab [4]
**Exclusion based on small vessel disease markers**							
Microhemorrhages	>1	Not specified [7]); >4 [8]	>2 [26]; >5 micro-hemorrhages + superficial siderosis [11]	>4	>4	>4	>4
Cortical superficial siderosis	Not specified	Not specified [7,8]	Not specified [26]; >5 micro-hemorrhages + superficial siderosis [11]	Yes	Yes	Yes	>1
White matter changes	Not specified	Not specified [7,8]	Extensive/ Confluent [26]; Fazekas score 3 [11]	Not specified	Diffuse involvement	Severe	Severe
**Exclusion based on ischemic or hemorrhagic stroke**							
History of clinical stroke	Yes	Not specified [7,8]	Yes [26]; Yes, within 1 year [11]	Yes	Yes, within 1 year	Yes, within 1 year	Not specified
Cortical infarcts on imaging	>1 cm^3^	Not specified [7,8]	Not specified [26]; territorial infarct > 1 cm^3^ [11]	Yes	>1.5 cm	Yes	Not specified
Lacunar infarcts on imaging	>1	Not specified [7,8]	>1 [26]; >2 [11]	Not specified	>1	Multiple	Not specified
ICH on imaging	>1 cm^3^	Not specified [7,8]	Not specified [11,26]	Yes	Yes	Yes	>1 cm
**Exclusion based on antithrombotic use**							
Aspirin use	Allowed	Not specified [7,8]	Not specified [26]; Allowed [11]	Not specified	Allowed	Allowed	Allowed
Other antiplatelet use	Clopidogrel and dipyridamole allowed	Not specified [7,8]	Not specified [26]; Allowed [11]	Not specified	Excluded	Allowed	Allowed
Anticoagulant use	Excluded	Not specified [7,8]	Not specified [26]; Excluded [11]	Not specified	Excluded	Allowed	Allowed

## Data Availability

Not applicable.
